# Gene therapy: advances, challenges and perspectives

**DOI:** 10.1590/S1679-45082017RB4024

**Published:** 2017

**Authors:** Giulliana Augusta Rangel Gonçalves, Raquel de Melo Alves Paiva

**Affiliations:** 1 Hospital Israelita Albert Einstein, São Paulo, SP, Brazil.

**Keywords:** Gene therapy, Genetic Vectors, Gene transfer, horizontal, CRISPR-Cas9, CAR-T cell, Genetic therapy, Clustered regularly interspaced short palindromic repeats, Terapia gênica, Vetores genéticos, Transferência genética horizontal, CRISPR-Cas9, CAR-T cell, Terapia genética, Repetições palindrômicas curtas agrupadas e regularmente espaçadas

## Abstract

The ability to make site-specific modifications to the human genome has been an objective in medicine since the recognition of the gene as the basic unit of heredity. Thus, gene therapy is understood as the ability of genetic improvement through the correction of altered (mutated) genes or site-specific modifications that target therapeutic treatment. This therapy became possible through the advances of genetics and bioengineering that enabled manipulating vectors for delivery of extrachromosomal material to target cells. One of the main focuses of this technique is the optimization of delivery vehicles (vectors) that are mostly plasmids, nanostructured or viruses. The viruses are more often investigated due to their excellence of invading cells and inserting their genetic material. However, there is great concern regarding exacerbated immune responses and genome manipulation, especially in germ line cells. *In vivo* studies in in somatic cell showed satisfactory results with approved protocols in clinical trials. These trials have been conducted in the United States, Europe, Australia and China. Recent biotechnological advances, such as induced pluripotent stem cells in patients with liver diseases, chimeric antigen receptor T-cell immunotherapy, and genomic editing by CRISPR/Cas9, are addressed in this review.

## RESUMO

A habilidade de fazer modificações pontuais no genoma humano tem sido o objetivo da medicina desde o conhecimento do DNA como unidade básica da hereditariedade. Entende-se terapia gênica como a capacidade do melhoramento genético por meio da correção de genes alterados (mutados) ou modificações sítio-específicas, que tenham como alvo o tratamento terapêutico. Este tipo de procedimento tornou-se possível por conta dos avanços da genética e da bioengenharia, que permitiram a manipulação de vetores para a entrega do material extracromossomal em células-alvo. Um dos principais focos desta técnica é a otimização dos veículos de entrega (vetores) que, em sua maioria, são plasmídeos, nanoestruturados ou vírus − sendo estes últimos os mais estudados, devido à sua excelência em invadir as células e inserir seu material genético. No entanto, existe grande preocupação referente às respostas imunes exacerbadas e à manipulação do genoma, principalmente em linhagens germinativas. Estudos em células somáticas *in vivo* apresentaram resultados satisfatórios, e já existem protocolos aprovados para uso clínico. Os principais *trials* têm sido conduzidos nos Estados Unidos, Europa, Austrália e China. Recentes avanços biotecnológicos empregados para o aprimoramento da terapia gênica, como células-tronco pluripotentes induzidas em pacientes portadores de doenças hepáticas, imunoterapia com células T do receptor do antígeno quimera e edição genômica pelos sistema CRISPR/Cas9, são abordados nesta revisão.

## INTRODUCTION

In 1991, James Watson declared that “many people say they are worried about the changes in our genetic instructions. But these (genetic instructions) are merely a product of evolution, shaped so we can adapt to certain conditions which might no longer exist. We all know how imperfect we are. Why not become a little better apt to survive?”.^(^ ^1^ ^)^ Since the beginning, humans understand that the peculiar characteristics of the parents can be transmitted to their descendents. The first speculation originated from the ancient Greek students, and some of these theories continued for many centuries. Genetic-scientific studies initiated in the early 1850s, when the Austrian monk, Gregor Mendel, in a series of experiments with green peas, described the inheritance pattern by observing the traces that were inherited as separate units, which we know today as genes. Up until 1950, little was known as to the physical nature of genes, which was when the American biochemist, James Watson, and the British biophysicist, Francis Crick, developed the revolutionary model of the double strand DNA. In 1970, researchers discovered a series of enzymes that enabled the separation of the genes in predetermined sites along the DNA molecule and their reinsertion in a reproducible manner. These genetic advances prepared the scenario for the emergence of genetic engineering with the production of new drugs and antibodies, and as of 1980, gene therapy has been incorporated by scientists.^(^ ^2^ ^,^ ^3^ ^)^

In this review, we cover gene therapy, the different methodologies of genetic engineering used for this technique, its limitations, applications, and perspectives.

### Gene therapy

The ability to make local modificiations in the human genome has been the objective of Medicine since the knowledge of DNA as the basic unit of heredity. Gene therapy is understood as the capacity for gene improvement by means of the correction of altered (mutated) genes or site-specific modifications that have therapeutic treatment as target. Further on, diffrent strategies are described, which are often used for this purpose.^(^ ^4^ ^)^

Currently, gene therapy is an area that exists predominantly in research laboratories, and its application is still experimental.^(^ ^5^ ^)^ Most trials are conducted in the United States, Europe, and Australia. The approach is broad, with potential treatment of diseases caused by recessive gene disorders (cystic fibrosis, hemophilia, muscular dystrophy, and sickle cell anemia), acquired genetic diseases such as cancer, and certain viral infections, such as AIDS.^(^ ^3^ ^,^ ^6^ ^)^

One of the most often used techniques consists of recombinant DNA technology, in which the gene of interest or healthy gene is inserted into a vector, which can be a plasmidial, nanoestrutured, or viral; the latter is the most often used due to its efficiency in invading cells and introducing its genetic material. On table 1, a few gene therapy protocols are summarized, approved and published for clinical use, exemplifying the disease, the target, and the type of vector used.^(^ ^3^ ^)^

**Table 1:** Gene therapy protocols

Disease	Objective	Stem cells	Release mode	Countries with the protocol
Adenosine deaminase deficiency	Substitution of the adenosine deaminase deficiency	Blood	Retrovirus	Italy, Holland, and the United States
α 1-antitrypsin deficiency	Substitution of α 1-antitrypsin	Respiratory epithelium	Liposome	United States
AIDS	Inactivation of the HIV-presenting antigen	Blood and bone marrow	Retrovirus	United States
Cancer	Improvement of immune function	Blood, bone marrow, and tumor	Retrovirus, liposome, electroporation, and cell-mediated transfer	Austria, China, France, Germany, Italy, Holland [Netherlands], and the United States
Cancer	Tumor removal	Tumor	Retrovirus, non-complexed DNA, cell-mediated transfer	United States
Cancer	Chemoprotection	Blood and bone marrow	Retrovirus	United States
Cancer	Stem cell marking	Blood, bone marrow, and tumor	Retrovirus	Canada, France, Sweden and United States
Cystic fibrosis	Enzymatic substitution	Respiratory epithelium	Adenovirus and liposome	England and the United States
Familial hypercholesterolemia	Substitution of low-density lipoprotein receptors	Liver	Retrovirus	United States
Fanconi anemia	Complement C gene release	Blood and bone marrow	Retrovirus	United States
Gaucher Disease	Glucocerebrosidase substitution	Blood and bone marrow	Retrovirus	United States
Hemophilia B	Factor IX substitution	Skin fibroblasts	Retrovirus	China
Rheumatoid arthritis	Cytokine release	Synovial membrane	Retrovirus	United States

Source: Adapted from Misra S. Human gene therapy: a brief overview of the genetic revolution. J Assoc Physicians India. 2013;61(2):127-33. Review.^(3)^

Although several protocols have been successful, the gene therapy process remains complex, and many techniques need new developments. The specific body cells that need treatment should be identified and accessible. A way to effectively distribute the gene copies to the cells must be available, and the diseases and their strict genetic bonds need to be completely understood.^(^ ^3^ ^)^ There is also the important issue of the target cell type of gene therapy that currently is subdivided into two large groups: gene therapy of the germline^(^ ^7^ ^)^ and gene therapy of somatic cells.^(^ ^8^ ^)^ In germline gene therapy, the stem cells, *e.g.*, with the sperm and egg, are modified by the introduction of functional genes, which are integrated into the genome. The modifications are hereditary and pass on to subsequent generations. In theory, this approach should be highly effective in the fight against genetic and hereditary diseases. Somatic cell gene therapy is when therapeutic genes are transferred to a patient’s somatic cells. Any modification and any effects are restricted only to that patient and are not inherited by future generations.

### Gene therapy process: release of the gene

In gene therapy, a normal gene is inserted into the genome to replace an abnormal gene responsible for causing a certain disease. Of the various challenges involved in the process, one of the most significant is the difficulty in releasing the gene into the stem cell. Thus, a molecular carrier called a “vector” is used to release the gene, which needs to be very specific, display efficiency in the release of one or more genes of the sizes necessary for clinical applications, not be recognized by the immune system, and be purified in large quantities and high concentrations so that it can be produced and made available on a large scale. Once the vector is inserted into the patient, it cannot induce allergic reactions or inflammatory process; it should increase the normal functions, correct deficiencies, or inhibit deleterious activities. Furthermore, it should be safe not only for the patient, but also for the environment and for the professionals who manipulate it. Finally, the vector should be capable to express the gene, in general, for the patient’s entire life.^(^ ^3^ ^,^ ^9^ ^)^

Although the efficacy of viral vectors is confirmed, recently some studies demonstrated that the use of these carriers presented with several limitations. The presence of viral genetic material in the plasmid is a strong aggravating factor, since it can induce an acute immune response, besides a possible oncogenic transformation. Currently, there are two main approaches for genetic modifications of the cells, namely: virus-mediated (Table 2) and via physical mechanisms, from preparations obtained by advanced nanotechnology techniques.^(^ ^5^ ^)^ Within this context, included are polymers that form networks that capture a gene and release its load when they penetrate the cells, such as DNA microinjections,^(^ ^10^ ^)^ cationic polymers,^(^ ^11^ ^)^ cationic liposomes,^(^ ^12^ ^,^ ^13^ ^)^ and particle bombardment.^(^ ^14^ ^)^

**Table 2:** Viral vectors for gene therapy

	Retrovirus	Lentivirus	Herpes virus	Adenovirus	Adenoassociated	Plasmid
Provirus	RNA	RNA	RNA	DNA	DNA	DNA
Capacity	~9 kB	~10 kB	>30 kB	~30 kB	~4.6 kB	Unlimited
Integration into the recipient genome	Yes	Yes	Yes	No	Extremely rare	No
Duration of transgene expression	Long	Long	Transient	Transient	Long in post-mitotic cells	Transient
Preexisting immunity in the recipient	No	No	Yes	Yes	Yes	No
Adverse effects	Insertional mutagenesis	Insertional mutagenesis	Inflammatory response	Inflammatory response	Mild inflammatory response	No
Germline transmission	May occur	Yes	No	No	May occur	No

Source: Modified from Linden R. Gene therapy: what it is, what it is not, and what it will be. Estud Av. 2010;24(70):31-69.^(5)^

Each exogenous material introduction technique differs from the other and depends on the type of application proposed. Some are more efficient, others more apt to carry large genes (>10kB) and integrate with the genome, allowing a permanent expression.^(^ ^1^ ^)^

### Gene therapy and hematopoietic stem cells

**Figure 1:**
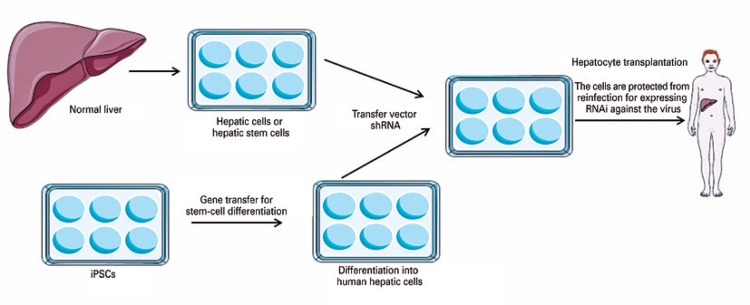
Combination of stem cells and gene therapy

Source: Adapted from Kay MA. State-of-the-art gene-based therapies: the road ahead. Nat Rev Genet. 2011;12(5):316-28. Review.^(^^15^^)^

Hematopoietic stem cells have become ideal targets for gene transfer due to the high potential for longevity and the capacity for self-renovation. One example of this combination of gene therapy and stem cells would be the production of gene transfer vectors for the creation of induced pluripotent stem cells (iPS), in order to generate the differentiation of the iPS and afford an additional phenotype from this differentiated derived cell. Patients with chronic liver disease and infection by the hepatitis virus (*e.g.*, hepatitis B virus and hepatitis C virus), which require a liver transplant, may be likely to undergo the hepatic transplantation of mature hepatocytes or those derived from iPS.^(^ ^15^ ^)^ Not only the transfer of genes might be needed to convert stem cells into hepatocytes; since the transplanted cells are susceptible to reinfection by the hepatitis virus, the transfer of a vector that encodes a short hairpin RNA directed against the virus would provide the transferred cells with resistance or ‘immunity’ to reinfection. Resistant cells can repopulate the liver over time and restore normal hepatic function (Figure 1).^(^ ^15^ ^)^

shRA: short hairpin RNA; iPS: induced pluripotent stem cells.

### Therapy with T cells of the recipient of a chimeric antigen

Chimeric antigen recipient T (CAR-T) cell therapy is a type of immunotherapy that involves manipulation/reprogramming of immune cells (T lymphocytes) of the patients themselves, in order to recognize and attack the tumor T cells. Initial advancement in the design of the first CAR generation, by Eshhar et al.,^(^ ^16^ ^)^ was marked by the fusion of a single chain fragment variable (scFv) to a transmembrane domain and an intracellular signaling unit: chain CD3 zeta.^(^ ^17^ ^,^ ^18^ ^)^ This design combined the active element of a well-characterized monoclonal antibody with a signaling domain, increasing the recognition of the tumor-specific epitope and the activation of T cells, without depending on molecules from the histocompatibility complex.

An improvement in the first generation of CAR was made by means of integrating co-stimulating molecules necessary for signal transduction. The stimulatory recipient most commonly used in this CAR generation is CD28. This recipient acts as a second activating event of the route, enabling a marked proliferation of T cells along with an increased expression of cytokines.^(^ ^19^ ^)^

The most recent generation of CAR incorporated the addition of a co-stimulatory domain addition to increase the CAR function. Co-stimulatory molecules as recipients of the tumor necrosis factor (CD134 or CD137) are required for this methodology. In summary, the most recent forms of CAR include scFv, the initial chain of CD3-ζ, along with the stimulatory chains of CD28 and CD134 or CD137.^(^ ^20^ ^)^

With the third CAR generation, Zhong et al., demonstrated an improvement in T cell activation of the Akt route (protein kinase B), which regulates the cell cycle. According to other studies, this last generation shows greater persistence of the T cells in comparison with the second generation of CAR.^(^ ^21^ ^)^

The most critical point of the adverse effects of CAR-T therapy is the identification of non-tumor cells that express the target epitope by CAR. Tumor antigens are molecules highly expressed in the tumor cells, but are not exclusive of these cells. For example, the CD19 antigen can be found in normal or malignant B cells, and the CAR design for the CD19 target in not capable of distinguishing them.^(^ ^20^ ^,^ ^22^ ^)^ Other common toxicity for CAR-T therapy (and many other types of immunotherapy for cancer) is the cytokine release syndrome (CRS). Activation of the immune system after CAR-T infusion can induce a rapid increase in the levels of inflammatory cytokines.^(^ ^20^ ^,^ ^23^ ^)^

New developments in the design of vectors and trials with CAR-T provide balance and reinforcement in safety for amplification of the clinical application. The progressive improvement in the CAR trials has already advanced, as was observed from the first to the third generation. Knowledge and experience acquired in the assessment of CAR-T toxicity will increase the success of the progressive improvements for future trials.

### CRISPR*-*Cas9

During the 1980’s, in the genome of *Escherichia coli*, a region was identified with an uncommon pattern, in which a highly variable sequence was intercalated by a repeated sequence with no known function. In 2005, it was assumed that the variable sequences were of extra-chromosomal origin, acting as an immune memory against phages and plasmids, starting the then unknown CRISPR system (Clustered Regularly Interspaced Short Palindromic Repeats*)* and Cas (Associated Proteins), that shines since 2012 as one of the primary biotechnological tools for gene edition.^(^ ^24^ ^)^ Originating in the immune-adaptive system of procaryontes, this mechanism recognizes the invading genetic mateiral, cleaves it into small fragments, and integrates it into its own DNA. In a second infection by the same agent, the following sequence occurs: transcription of the CRISPR locus, RNAm processing, and creation of small fragments of RNA (crRNAs) that form complexes with the Cas proteins, and these recognize the alien nucleic acids and finally destroy them.^(^ ^24^ ^)^

**Figure 2:**
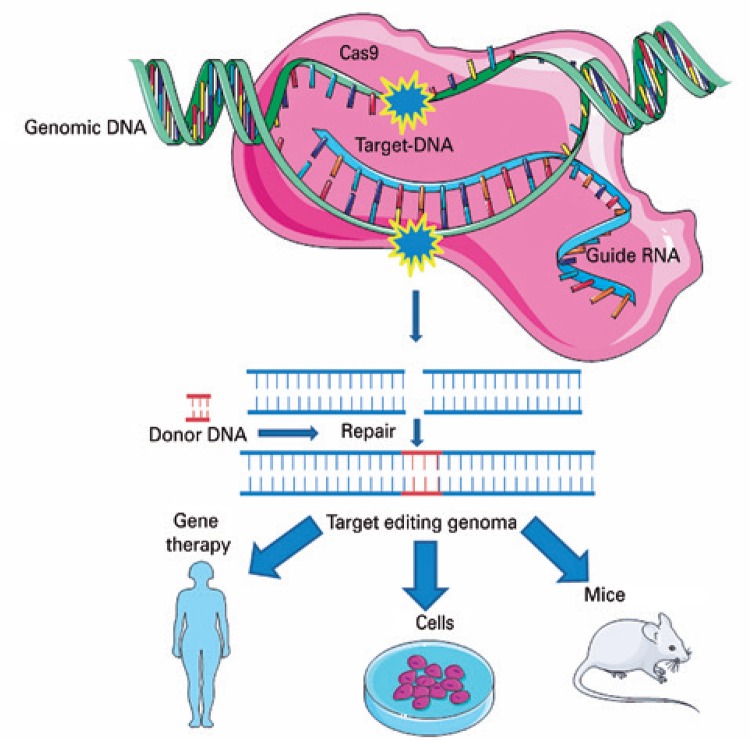
CRISPR Cas-9 system. The technique involves basically three molecules: one nuclease (generally wild type Cas-9 of *Streptococcus pyogenes*), an RNA guide (known as single guide RNA), and the target (frequently the DNA)

Source: Modified from Vieira GV, Cecílio NT, Arruda LM, Sales KU. Visão geral do mecanismo básico de ação. In: Pereira TC, organizador. Introdução à técnica de CRISPR. Ribeirão Preto: Cubo; 2016. Cap. 2. p. 54.^(^^27^^)^

Based on this natural mechanism, the CRIPSR technique was developed enabling editing of the target-specific DNA sequences of the genome of any organism by means of basically three molecules: nuclease (Cas9), responsible for cleavage of the double-strand DNA; an RNA guide, which guides the complex to the target; and the target DNA, as is shown in figure 2.^(^ ^25^ ^,^ ^26^ ^)^

Due to its simplicity and its precision when compared to other techniques (Zinc-Finger Nucleases, TALENs, and Gene Targeting), the CRISPR system arrives as a versitile tool that promotes the genetic editing by means of inactivation (knockout gene − KO), integration of exogenous sequences (knock-in)*,* and allele substitution, among others.^(^ ^27^ ^,^ ^28^ ^)^

The guide RNA hybridizes with the target DNA. Cas-9 recognizes this complex and should mediate cleavage of the DNA double strand and reparation in the presence of a (homologous) donor DNA. The result of this process is the integration of an exogenous sequence into the genome (knock-in) or allele substitution.

The rapid advancement of this new technology allowed the performance of translational trials in human somatic cells, using genetic editing by CRISPR. The first applications with a therapeutic focus already stood out in describing even the optimization steps of the delivery systems and specificity for the safety and efectiveness of the system.^(^ ^28^ ^,^ ^29^ ^)^

Researchers from the University of California and of Utah recently were successful in correcting the mutation of the hemoglobin gene, which originates sickle cell anemia. CD34+ cells from patients who are carriers of sickle cell anemia were isolated, edited by CRISPR-Cas9, and after 16 weeks, the results showed a reduction in the expression levels of the mutated gene and an increased gene expression of the wild type.^(^ ^29^ ^)^

The technology referred to is in use mainly in monogenic genetic pathologies, which, despite being rare, can reach about 10 thousand diseases already described.^(^ ^4^ ^)^ Phase 1 clinical trials are foreseen for 2017, as well as the appearance of companies geared toward the clinical use of this system.

### Ethical issues

The possibility of genetically modifying germlines has been the object of heated discussion in the field of science for a long time. Bioethics is always present when new techniques are created, in order to assess the risks of the procedure and the moral implications involved.

A large part of the scientific community approves genetic therapy in somatic cells, especially in cases of severe disorders, such as cystic fibrosis and Duchenne muscular dystrophy.

In 2015, however, Chinese researchers went beyond the moral issues and announced, for the first time, the genetic modification of embryonic cells using the CRISPR-Cas9 technique. Next, another Chinese group also reported the conduction of the same process done with the intention of conferring resistance to HIV by insertion of the CCR5 gene mutation. The genetic analysis showed that 4 of the 26 embryos were successfully modified. The result clearly reveals the need for improving the technique, alerting that, possibly, such trials could be previously tested in animal models.^(^ ^4^ ^,^ ^30^ ^)^

These recent publications rekindled the debate regarding genetic editing. On one side, the Japanese Ethics Committee declared that the manner in which the experiment was conducted was correct, since there had been approval by the local Ethics Committee for the study conducted, as well as the consent of the egg donors. In the United Kingdom, the first project for healthy human embryo editing was approved. On the other hand, American research groups remained conservative, reiterating their position of not supporting this type of experiment and declaring that they await improvement in the techniques and of the definitions of ethical issues.^(^ ^30^ ^)^

## COMMENT

Since the declaration of James Watson in 1991, in reference to the likely optimization of human genetics, gene therapy has advanced throughout the decades, whether by optimization of the types of vectors, by the introduction of new techniques, such as induced pluripotent stem cells in combination with current models of genetic editing (CRISPR-Cas9), and even by trials in germ cells, bringing with it the contradictory ethical and moral aspects that accompany the technique.

Local successes have already solidified the viability of treatments using gene therapy in clinical practice, as an alternative form for patients with congenital diseases or monogenic disorders and cancer, especially when the pharmacological or surgical interventions do not show good results.

The design of new experimental vectors, the increase in efficiency, the specificity of the delivery systems, and the greater understanding of the inflammatory response induction may balance the improvement of safety with the expansion of techniques in clinical applications. Yet the knowledge and experience acquired with the careful assessment of toxicity of these technologies also allow significant advances in the application of these methods.

Therefore, historically, gene therapy and the discovery of antibiotics and chemotherapy agents, or any new technology, need more clarifying preclinical studies. In the future, there is the promise of applying these techniques in several fields of Medicine and a greater percentage of clinical trials.
